# Adapting to COVID-19 at arch

**DOI:** 10.1192/j.eurpsy.2021.1532

**Published:** 2021-08-13

**Authors:** A. Selladurai, C. Goedhuis, J. Fehler

**Affiliations:** Addiction Psychiatry, CNWL, London, United Kingdom

**Keywords:** addictions, COVID-19, supervised consumption

## Abstract

**Introduction:**

The Addictions Recovery Community Hillingdon (ARCH) is a specialist addictions treatment service, providing a range of inteventions for substance use disorders. The onset of the COVID-19 pandemic required healthcare services to rapidly adapt clinical care in order to safeguard patients and staff from contracting the virus whilst managing clinical risk. Key changes were made to treatment pathways at ARCH.

**Objectives:**

1. Reduce face-to-face contact between patients and staff (including community pharmacists) 2. To get feedback from patients and staff about changes implemented

**Methods:**

To reduce face-to-face contact, we aimed to decrease the number of patients having supervised consumption of Opiate Substitute Treatment (OST). Furthermore, telephone consultations were encouraged for keyworking and reviews. Patients were randomly selected and interviewed about their experiences and focus groups were be completed with staff.

**Results:**

Supervised consumption of OST was reduced from 41.5% to 6%. Face-to-face appointments were significantly reduced and telephone consultations were introduced as standard. Telephone reviews became the standard method of contact for keyworking sessions and medical reviews. 53% of services whose interval between instalment collection of OST at community pharmacies was extended found it ‘easy’ or ‘very easy’ to adapt to. 61% of service users who had access virtual platforms finding it ‘easy’ or ‘very easy’ to access support. Focus groups of staff members revealed that stafff felt the changes in instalment collection of OST was positive for patients.

**Conclusions:**

ARCH implemented a number of changes to treatment pathways and inteventions to minimise the risk of virus transmission amongst patients and staff whilst managing clinical risk.

